# A biomechanical comparison between locked 3.5-mm plates and 4.5-mm plates for the treatment of simple bicondylar tibial plateau fractures: is bigger necessarily better?

**DOI:** 10.1007/s10195-013-0275-6

**Published:** 2013-11-26

**Authors:** Saqib Hasan, Omri B. Ayalon, Richard S. Yoon, Amit Sood, Ulises Militano, Mark Cavanaugh, Frank A. Liporace

**Affiliations:** 1 Division of Orthopaedic Trauma, Department of Orthopaedic Surgery, NYU Hospital for Joint Diseases, 301 E 17th Street, Suite 1402, New York, NY 10003 USA; 2 Division of Orthopaedic Trauma, Department of Orthopaedic Surgery, Rutgers New Jersey Medical School, Newark, NJ 07103 USA; 3 Division of Orthopaedic Trauma, Department of Orthopaedic Surgery, Orange Park Medical Center, Jacksonville, FL 32073 USA

**Keywords:** Tibial plateau, Biomechanical, Locked plating, Periarticular, Bicondylar

## Abstract

**Background:**

Evolution of periarticular implant technology has led to stiffer, more stable fixation constructs. However, as plate options increase, comparisons between different sized constructs have not been performed. The purpose of this study is to biomechanically assess any significant differences between 3.5- and 4.5-mm locked tibial plateau plates in a simple bicondylar fracture model.

**Materials and methods:**

A total of 24 synthetic composite bone models (12 Schatzker V and 12 Schatzker VI) specimens were tested. In each group, six specimens were fixed with a 3.5-mm locked proximal tibia plate and six specimens were fixed with a 4.5-mm locking plate. Testing measures included axial ramp loading to 500 N, cyclic loading to 10,000 cycles and axial load to failure.

**Results:**

In the Schatzker V comparison model, there were no significant differences in inferior displacement or plastic deformation after 10, 100, 1,000 and 10,000 cycles. In regards to axial load, the 4.5-mm plate exhibited a significantly higher load to failure (*P* = 0.05). In the Schatzker VI comparison model, there were significant differences in inferior displacement or elastic deformation after 10, 100, 1,000, and 10,000 cycles. In regards to axial load, the 4.5-mm plate again exhibited a higher load to failure, but this was not statistically significant (*P* = 0.21).

**Conclusions:**

In the advent of technological advancement, periarticular locking plate technology has offered an invaluable option in treating bicondylar tibial plateau fractures. Comparing the biomechanical properties of 3.5- and 4.5-mm locking plates yielded no significant differences in cyclic loading, even in regards to elastic and plastic deformation. Not surprisingly, the 4.5-mm plate was more robust in axial load to failure, but only in the Schatzker V model. In our testing construct, overall, without significant differences, the smaller, lower-profile 3.5-mm plate seems to be a biomechanically sound option in the reconstruction of bicondylar plateau fractures.

## Introduction

High-energy complex bicondylar fractures constitute a small subset of all tibial fractures, however, they present a significant challenge with regard to surgical effort and planning [1, 2]. The characteristic metaphyseal and articular comminution with concomitant violation of a tenuous soft tissue envelope entails an operatively demanding procedure with the potential for significant post-operative complications [3]. Historically, complications and poor results were seen in 20–70 % of this fracture subtype [3–5], providing an impetus for continuous evolution in treatment modalities. The controversy surrounding ideal management of these fractures has resulted in a variety of different fixation methods depending on specific fracture patterns, integrity of the soft-tissue envelope and bone quality. The goals of reconstruction of articular congruity and restoration of anatomic alignment and joint stability to allow early joint motion and weight bearing are balanced with the challenge of preserving the local biological environment. Traditional options for fixation include the use of bilateral buttress plates, a lateral buttress plate with a smaller posterior medial plate, hybrid external fixators and the more recent use of lateral locking plates.

Conventional dual plate osteosynthesis necessitates invasive dissection of a precarious soft tissue envelope with concomitant compression of the plate to bone with potential compromise of tenuous local periosteal vasculature. Complications of wound breakdown, deep tissue infection, compartment syndrome, delayed union, nonunion, secondary loss of reduction, peroneal palsies, hardware failure and arthrofibrosis have been well-documented throughout the literature resulting in great variability in achieving satisfactory outcomes [5–12]. Similarly, while the use of hybrid external fixators obviates the need for extensive surgical dissection, superficial and pin tract infections, osteomyelitis, septic arthritis, varus malalignment and loss of knee motion are among the many complications reported [13–16].

The emergence of locked plate technology for periarticular distal femur fracture fixation led to the design of a similar fixation construct for the proximal lateral tibia [17, 18]. Locking plates function as internal fixators with the locking screws creating a fixed angle construct providing angular stability. The fixed angle nature of the locked plate circumvents the need for additional medial stabilization, reducing the risk of injury to medial soft tissues [19]. Some literature has suggested that the use of locked plate technology to be biomechanically equivalent to the historical control of double plating [20–22], with the advantage of less soft tissue dissection. Numerous studies have reported successful outcomes with the use of single lateral locking plate fixation of complex bicondylar tibial plateau fractures [9, 20, 23–26]. Concerns arise when there is significant metaphyseal comminution for a long segment or with certain patterns of medial articular involvement that may not be adequately supported by the trajectory of fixation from a solely laterally based implant.

Previous biomechanical studies investigating fixation of complex bicondylar fractures have used the less invasive stabilization system (LISS) proximal tibia locking plates, a large fragment plate constructed from titanium with 5.0-mm locking screws [20, 21]. Newer proximal tibial locking plates have stainless steel implant options as well as different fragment sizes. To our knowledge, a biomechanical comparison of different sized proximal tibial locking plates in a simple proximal tibial fracture model has not been reported previously. The purpose of this biomechanical study was to compare 3.5- and 4.5-mm proximal tibia locking plates in order to determine the overall stability of fixation in a simulated bicondylar tibial plateau fracture (Schatzker V) and a simulated bicondylar tibial plateau fracture with meta-diaphyseal separation (Schatzker VI).

## Materials and methods

Ethical review board approval was not required due to the non-human subject nature of this study; all proper laboratory protocols were followed in the completion of this study. Because there is a wide variability in bone quality in cadaveric specimens, synthetic material was selected in order to standardize testing specimens. Advantages of composite tibial sawbones include less variability among specimens, ease of availability and handling and lack of degradation. There are several studies that show that the biomechanical properties of these simulated bones are equal to cadaveric tibias [27, 28]. Cristofolini and Viceconti [28] showed that the bending stiffness for composite tibias was similar to cadaver bone. The composite tibias were significantly stronger in torsional loading compared to cadaver bone, but in this study the tibias were not subject to such testing.

**Fig. 1:**
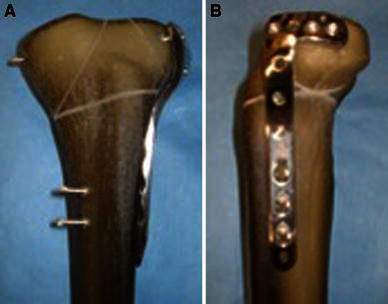
Bicondylar tibial plateau fracture model

Based on a previous model described by Horwitz et al. [29], Schatzker V and VI tibial plateau fractures were created in our composite tibial sawbones. These fractures were anatomically reduced under direct vision and then fixed with either a 3.5- or 4.5-mm locking plate by a fellowship-trained orthopedic trauma surgeon (FAL). A total of 24 (12 Schatzker V and 12 Schatzker VI) specimens were tested. In each group, six specimens were fixed with a 3.5-mm locked proximal tibia plate and six specimens were fixed with a 4.5-mm locking plate. Figure 1 depicts our bicondylar tibial plateau fracture model. Three locked subchondral screws were placed in the proximal aspect of the plate. A locked kickstand screw was placed in the lateral metaphysis into the subchondral bone in the medial tibial plateau. Two locked screws were placed into the shaft of the tibia in the distal aspect of the plate. The diaphyseal screws used in the saw bones, as seen in Fig. 1, were significantly longer than those normally used in vivo. This was done in order to show the trajectory of the diaphyseal screws and in previous studies have been utilized as such without compromising the study [30]. Our goal was to maintain the lever arm by maintaining two cortices of contact, which has been shown to diminish with only a unicortical screw purchase; as noted previously, this principle helps to maintain biomechanical stability for testing and does not change with excessive length as another contact point is not added [31].

The tibial composites were then mounted into a holding fixture using bone cement in neutral alignment. The bone/implant constructs were mounted on a Material Testing System (Instron Inc, Norwood, Massachusetts, USA) servo hydraulic testing machine. The constructs were ramp-loaded to 500 N at a rate of 100 N/s. The load was applied to the medial tibial plateau using a spherical loader. The medial tibial plateau has been shown by Horwitz et al. [29] to be the most unstable part of a bicondylar tibial plateau fracture model.

Each tibia was then cyclically loaded from 50 to 500 N for 10^5^ cycles at 3 Hz. Displacement measurements were taken at 10, 100, 1,000 and 10,000 cycles with and without the 500-N applied load. The constructs were then tested for ultimate tensile force. Axial compressive forces starting at 500 N were applied and increased until failure. Failure was defined as greater than 3 mm of displacement in the articular surface in the medial condyle. Medial tibial subsidence was measured using the actuator from the MTS machine. The testing protocol used was based on previous studies by Horwitz et al. [29] and Egol et al. [20].

Statistical analysis of the stiffness and displacement values of the paired specimens was performed using Student’s *t* tests. A *P* value less than 0.05 was considered statistically significant.

## Results

**Fig. 2:**
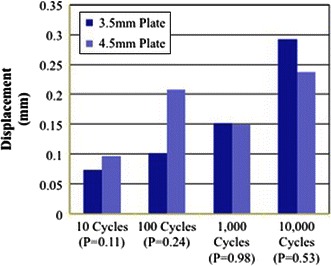
Schatzker V: plastic deformation, no significant differences noted

The results of cyclic loading with the 500-N load applied to the medial condyle in a Schatzker V simulated model showed there was no significant difference in inferior displacement of the medial fragment between the 3.5- and 4.5-mm fragment plates after 10, 100, 1,000 and 10,000 cycles (Table 1). Elastic deformation was not statistically different between the two plates. The results of the cyclic loading without the 500-N load applied showed there was no significant difference in inferior displacement of the medial fragment between the 3.5- and 4.5-mm fragment plates at the measured cycles (Fig. 2). Plastic deformation was not statistically different between the two plates, although there was a trend towards increasing deformation of 3.5-mm plates at greater than 1,000 cycles in the Schatzker V model.

**Table 1:** Schatzker V: elastic deformation, no significant differences noted

	Ramp loading displacement (mm)	Displacement (10 cycles) (mm)	Displacement (100 cycles) (mm)	Displacement (1,000 cycles) (mm)	Displacement (10,000 cycles) (mm)
3.5-mm plate	0.236 ± 0.04	0.258 ± 0.02	0.507 ± 0.08	0.684 ± 0.1	0.821 ± 0.2
4.5-mm plate	0.233 ± 0.02	0.268 ± 0.02	0.536 ± 0.1	0.639 ± 0.1	0.720 ± 0.1

**Fig. 3:**
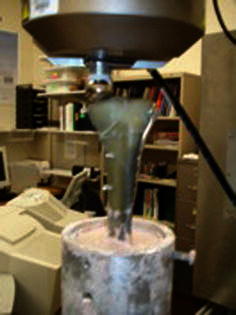
Biomechanical testing construct

**Fig. 4:**
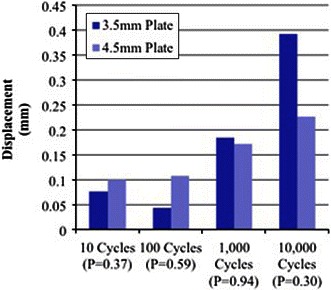
Schatzker VI: plastic deformation, no significant differences noted

The results of cyclic loading with the 500-N load applied to the medial condyle in a Schatzker VI simulated model showed there was no significant difference in inferior displacement of the medial fragment between the 3.5- and 4.5-mm fragment plates after 10, 100, 1,000 and 10,000 cycles (Fig. 3). Elastic deformation was not statistically different between the two plates (Tables 1, 2). The results of the cyclic loading without the 500-N load applied showed there was no significant difference in inferior displacement of the medial fragment between the 3.5-mm and 4.5-mm fragment plates at the measured cycles (Fig. 4). Similarly to the Schatzker V model, plastic deformation was not statistically different between the two plates, although there was a trend towards increasing deformation of the 3.5-mm plates at greater than 1,000 cycles.

**Table 2:** Schatzker VI: elastic deformation, no significant differences noted

	Ramp loading displacement (mm)	Displacement (10 cycles) (mm)	Displacement (100 cycles) (mm)	Displacement (1,000 cycles) (mm)	Displacement (10,000 cycles) (mm)
3.5-mm plate	0.231 ± 0.02	0.256 ± 0.01	0.629 ± 0.2	0.823 ± 0.2	1.03 ± 0.2
4.5-mm plate	0.246 ± 0.04	0.259 ± 0.02	0.669 ± 0.4	0.800 ± 0.4	0.880 ± 0.4

**Fig. 5:**
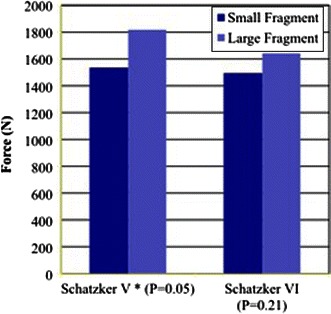
Load to failure, significantly higher load to failure in the Schzatker V model (*P* = 0.05), and a trend noted in the Schaztker VI model

With regard to axial loading, the load to failure for the Schatzker V model fixed with a 4.5-mm plate was significantly higher (*P* = 0.05) than the load to failure with for the model fixed with the 3.5-mm plate. The load to failure for the Schatzker VI model fixed with a 4.5-mm plate was higher than the load to failure for the model fixed with the 3.5-mm plate, although these results were not statistically significant (*P* = 0.21) (Fig. 5).

## Discussion

The evolution of locking-plate technology has led to newly designed, anatomically preshaped plates for different fractures, such as proximal humerus, proximal and distal femur and proximal and distal tibia [32, 33]. The purpose of this study was to compare 3.5- and 4.5-mm proximal tibia locking plates in order to determine the overall stability of fixation in a simulated bicondylar tibial plateau fracture (Schatzker V) and a simulated bicondylar tibial plateau fracture with meta-diaphyseal separation (Schatzker VI). The hypothesis tested was that there would be no difference in biomechanical strength between the two plates used in either fracture model.

Based on the data from ramp loading, cyclic loading and load to failure, the use of a 3.5-mm locked plate is not biomechanically different (to a statistically significant degree) from a 4.5-mm locked plate in the treatment of simple pattern bicondylar tibial plateau fractures. Previous biomechanical research on complex bicondylar tibial plateau fractures has focused on the use of the LISS proximal tibia locking plates; a large fragment plate constructed from titanium with 5.0-mm locking screws.

Egol et al. [20] compared the use of the LISS plate with dual plating using a lateral plate and anteromedial anti-glide plate. No difference was found between the constructs when the specimens were axially loaded to 500 N. After cyclic loading there was a significant difference found in the average displacement with the 500-N load applied, however, once the load was removed there was no longer a significant difference in displacement. The authors felt the difference in cyclic loading was due to the lower modulus of elasticity of the LISS plate as compared to the stainless steel implants used in the dual plating construct.

Even though the authors found more displacement with the locked plate, they felt the fixation was still sufficient to adequately treat a bicondylar tibial plateau fracture. There was also a clinical aspect to this study in which 38 patients with Schatzker V and VI tibial plateau fractures were treated with a LISS plate. Thirty-six of 38 (94 %) patients united at 4 months with no loss of fixation or infection. Egol et al. felt that the micromotion that occurred may allow secondary bone healing and explain the callus found in the metaphyseal region seen on the X-rays in the clinical part of this study. The locked plate used in our current study was stainless steel and therefore more rigid than the LISS plate.

Gosling et al. [21] also compared a single lateral locked plate to dual plating with a lateral and anteromedial anti-glide plate. They looked at the plastic (non reversible) and elastic (reversible) deformation at four different loads (400, 800, 1,200 and 1,600 N). The plastic deformation was the amount of subsidence present after the load was removed from the specimen while the elastic deformation was the amount of subsidence with the load in place.

They concluded that the maximal subsidence found in the specimens was mainly reversible or elastic and depended on the fixation technique with the locked plate group having a significantly higher maximal subsidence. The amount of irreversible or plastic deformation did not differ between the two fixation methods. The authors felt that this difference was due to a lack of interfragmentary compression in the locked plate group. In the locked plate group the meta-diaphyseal gap remained in the specimens. The authors also stated that the higher amount of reversible motion may be desirable because this micromotion may enhance fracture callus formation. It was concluded that the locked plates had similar amounts of plastic deformation compared to a dual plating construct with less soft tissue stripping and therefore might be a better construct in the treatment of these complex fractures.

The testing performed in their study only consisted of 20 cycles. The authors recommended further testing to include load to failure and fatigue testing. These additional testing parameters were carried out in the present study and further support the use of locked plates in the treatment of these complex fractures.

We must remember that the use of locking (fixateur interne) plating simulates the mechanical properties of external fixation. Our study was successful in showing that a 4.5-mm locked proximal tibial plate may not always be required for treatment of tibia plateau Schatzker V and VI fractures when a simple fracture pattern exists in non-osteopenic bone that would allow true load-sharing between the implant and the bone. Use of the stouter plate may be supplanted by the 3.5-mm plate, as its use was shown to provide adequate and comparable fixation in our fracture model.

In Fig. 4, which depicts the displacement of both plates, the overall behavior of each is different, though this difference is not statistically different. The 3.5-mm plate exhibits a major initial displacement with progression to a linear rate of displacement after 1,000 cycles. The 4.5-mm plate, however, displays a more uniform linear rate of displacement throughout. This may be due to the fact that the thinner plate requires fewer initial cycles than the thicker plate to displace initially, with similar performances of both plates later on in load cycle progression.

In Fig. 2, the trends of each plate are slightly different. The 3.5-mm plate displays a more linear trend of displacement, while the 4.5-mm plate displays a more erratic one. This possibly can be explained by slight variations in screw placement between the two plate types. Also, the measurements of displacement were taken at time points remote from one another; a graph with significantly more data points would likely reflect a more accurate trend of the results.

Our findings should be applied to clinical settings reflective of the study parameters, specifically, Schatzker V and VI fractures with minimal or no comminution or fracture gapping along with adequate bone quality. As expected, the 3.5-mm plates displayed a trend toward greater plastic and elastic deformation as compared to the 4.5-mm plates.

This phenomenon may help explain the lack of significant difference between plate types in load to failure of both constructs. This seemingly inconsequential plastic and elastic deformation in the 3.5-mm plate may allow some compression across the large, non-comminuted fracture fragments, thus providing secondary stability which the more rigid 4.5-mm counterpart lacks. As stated before, this phenomenon would not apply to comminuted fractures in patients with poor bone quality and unfavorable healing biology.

Additionally, the more liberal use of the 3.5-mm locked proximal tibia plate has an advantage of being less prominent than its counterpart, thus helping to avoid potential irritation and future wound healing complications classically associated with such implants [23, 25, 26, 34]. Also, the small fragment option allows more points of subchondral fixation which may be able to be positioned more proximally and allow a greater buttress in scenarios with articular comminution.

As with all biomechanical studies, we have some limitations. Our constructs were tested using synthetic tibias without soft tissue attachments. The biomechanical properties of the synthetic tibias are similar to that of cadaveric and human bone, however they are not human specimens [27, 28]. The specimens were tested with the load applied to the medial tibial plateau. This type of loading is not entirely physiologic and does not reproduce the complex loading of a tibial plateau during gait, although it does accomplish testing the greatest lever arm of the construct. Lastly, a gap model was not created, so the results of this study cannot be applied to comminuted fractures.

In summary, in the correct clinical setting, a 3.5-mm locked proximal tibial plate may be used with comparable biomechanical strength when a 4.5-mm plate would have otherwise been used. Offering a lower-profile option with comparable strength, especially in the setting of little metaphyseal comminution and/or gapping, may prove favorable in these settings.
